# Between leaves and lives: Maternal mental health among tea garden workers in Bangladesh and its association with children’s behavioural difficulties

**DOI:** 10.1017/gmh.2026.10251

**Published:** 2026-06-15

**Authors:** Murad Ansary, Afsana Sultana, Maria Ferdousi, Md Saifur Rahman, Monaemul Islam Sizear, Kamal Uddin Ahmed Chowdhury, M Tasdik Hasan

**Affiliations:** 1 Clinical Psychology, University of Rajshahi, Rajshahi, Bangladesh; 2 Psycure Organization, Dhaka, Bangladesh; 3 Microbiology, Bangladesh University of Health Sciences, Dhaka, Bangladesh; 4 Public Health Foundation Bangladesh, Dhaka, Bangladesh; 5 Department of Clinical Psychology, University of Dhaka, Dhaka, Bangladesh; 6 Monash University, Melbourne, Australia

**Keywords:** maternal mental health, tea garden workers, child well-being, mixed-methods, psychological well-being

## Abstract

Despite growing recognition of the intergenerational effects of maternal mental health, evidence from tea garden communities remains limited. This study assessed the mental health status of working mothers in tea gardens of Moulvibazar, Bangladesh, examined its association with children’s well-being, and explored contextual factors shaping maternal mental health. A convergent cross-sectional mixed-methods study was conducted. Using a purposive and stratified random sampling technique, the quantitative data were collected from 451 working mothers with at least one child aged 2–7 years. Mothers’ psychological well-being was assessed using the Depression, Anxiety, Stress Scale-21 and World Health Organisation–Five scales, while children’s behavioural and emotional well-being was measured using the Strengths and Difficulties Questionnaire (SDQ). The qualitative component included 11 key informant interviews and two focus group discussions (FGDs) from Moulvibazar, Bangladesh. Quantitative data were analysed using descriptive and inferential statistics, and qualitative data were thematically analysed. A substantial proportion of mothers reported high levels of psychological distress, with 32.4%, 29.9% and 31.7% experiencing moderate to extremely severe depression, anxiety and stress, respectively, while 33.9% had poor well-being. Age was significantly associated with stress (*p* = 0.014) and overall well-being (*p* = 0.002), whereas no significant association was observed between childcare arrangements and anxiety (*p* = 0.171). Among children, 87.8% were classified as having abnormal SDQ scores based on maternal report. Addressing maternal mental health through improved working conditions, accessible childcare and targeted mental health interventions is critical for breaking cycles of intergenerational vulnerability.

## Impact statement

Millions of women around the world work in tea gardens and similar labour-intensive industries under conditions of chronic poverty, physical exhaustion and social marginalisation. Yet, their mental health has received almost no systematic research attention. This study brings that invisibility into focus. By listening directly to working mothers in the tea gardens of Bangladesh, through surveys and interviews, this research reveals that psychological distress is not an individual failing but a predictable consequence of structurally unjust working and living conditions. Low wages that cannot sustain a family, no toilets in the fields, biometric attendance systems that punish illness and the absence of childcare during an 8-h shift are not peripheral issues, they are the architecture of distress.

What makes these findings significant is their reach beyond the mothers. Children growing up in households where mothers report depression and anxiety are more likely to show behavioural and emotional difficulties, a pattern this study documents and gives voice to through the words of mothers, supervisors and community leaders. This is a pattern consistent with intergenerational disadvantage, and the structural conditions that produce it are modifiable.

The implications extend well beyond Bangladesh. Tea garden and plantation communities exist across South and Southeast Asia, Sub-Saharan Africa and Latin America and the structural conditions documented here, low wages, physical hazard, gender-based exploitation, absent childcare and stigmatised mental illness, are common across them. This study offers a model for how to research marginalised occupational communities using methods that honour both numbers and lived experience.

For policymakers, the message is practical: extended childcare, fairer wages, adequate sanitation and workplace safety are not luxuries; they are mental health interventions. For NGOs and garden management, the study identifies the leverage points, supportive supervisors, functioning village councils, and community awareness, that already protect women’s well-being and can be strengthened.

## Introduction

The tea industry in Bangladesh employs hundreds of thousands of workers, with estimates suggesting around 300,000 people, of whom more than 75% are women, making women the majority of the workforce engaged in labour-intensive roles such as tea plucking and field work (Ahmed et al., 2022). Women employed in these tea gardens experience a complex intersection of socio-economic and structural challenges that substantially affect their mental well-being. These challenges include low and irregular wages, physically demanding labour, unsafe working conditions, limited access to healthcare and entrenched gender discrimination within institutional and social systems (Auntu, 2021; Nowshin et al., 2021; Majumder and Chowdhury, 2024). The majority of tea garden workers belong to indigenous or other marginalised ethnic groups, experience social exclusion, have low levels of formal education and lack access to formal psychosocial support services (Faruk et al., 2021a,b; Faruk and Hasan, 2022). Many reside in geographically isolated and socio-economically deprived communities, further compounding their vulnerability (Hassan, 2014; Gupta et al., 2025; Islam and Al-Amin, 2025).

Women in these communities often shoulder a dual burden of paid labour and unpaid domestic and caregiving responsibilities. This cumulative workload increases psychological distress and heightens vulnerability to common mental health conditions, particularly depression and anxiety (Baumgartner et al., 2021). Existing evidence suggests that women facing economic hardship and high caregiving demands are disproportionately affected by mental health problems (Masud Ahmed, 2001; Patel et al., 2018; Cabezas-Rodríguez et al., 2021).Despite this evidence, there is a paucity of research examining mothers engaged in physically demanding, low-wage labour. The nature of employment itself may contribute to differential mental health risks (Ahn et al., 2019). Given the substantial socio-economic disparities between women engaged in manual labour and those in office-based employment, the risk and protective factors influencing mental health are likely to differ markedly across these groups (Jalil and Oakkas, 2018; Cabezas-Rodríguez et al., 2021). Poor maternal mental health in labour-intensive settings not only undermines women’s quality of life but also has profound implications for their children’s well-being (Xu and Xu, 2024).

There is growing recognition of the intergenerational transmission of mental distress, with maternal mental health playing a critical role in shaping child well-being. Children under 5 years of age whose primary caregivers experience untreated mental health conditions are at increased risk of developmental delays, emotional dysregulation, impaired social functioning and poorer physical health outcomes (Walker et al., 2007). Early childhood represents a sensitive period for emotional, cognitive and social development, during which maternal mental well-being is a key determinant of the child’s caregiving environment (Britto et al., 2017).

Children growing up in tea garden communities are typically exposed to persistent poverty and material deprivation, which normalises parental, particularly maternal, labour from an early age. Limited access to quality education, adequate nutrition and basic necessities significantly affects children’s physical, emotional and social development (Dijendra Chandra Acharja et al., 2025; Jamilur Rahman Saikat, 2025). While maternal employment may contribute to household survival, it can also pose risks to children’s psychological and physical health when accompanied by maternal mental distress (Hosokawa and Katsura, 2021). Empirical evidence indicates that maternal mental health is closely associated with socio-economic deprivation and is a strong predictor of child developmental outcomes (Okelo et al., 2025).

Drawing on the social determinants of health and the stress process model (Pearlin et al., 1981), this study conceptualises maternal mental health in tea garden communities as shaped by three interacting structural domains. First, occupational precarity, comprising low and irregular wages, physically hazardous working conditions, coercive attendance and quota systems and insecure employment status, constitutes a primary source of chronic psychological stress for working mothers. Second, household burden, comprising the dual demands of paid labour and unpaid domestic and caregiving responsibilities, compounded by limited spousal support and restricted autonomy, amplifies this occupational stress by reducing recovery time, increasing role conflict and limiting help-seeking. Third, childcare arrangements, specifically the absence of adequate, accessible and affordable childcare during working hours, function as both an independent stressor and a mediating condition: mothers unable to secure safe childcare for their children carry the additional psychological burden of worry about their children’s safety and well-being during the working day. Together, these three domains are hypothesised to produce elevated rates of depression, anxiety and poor psychological well-being among working mothers in tea garden communities. A second pathway then links maternal mental health to child outcomes: mothers experiencing untreated depression and anxiety have reduced capacity for responsive, attentive parenting, which in turn increases children’s exposure to emotional dysregulation, inconsistent care and psychosocial risk, manifesting as behavioural and emotional difficulties detectable through instruments such as the SDQ (Walker et al., 2007; Okelo et al., 2025). This study tests this conceptual model empirically in a population for which no prior evidence exists.

Identifying the factors that influence the mental health of working mothers in tea garden settings is critical for the development of effective, contextually appropriate interventions. However, there remains a significant gap in the literature, particularly studies that examine both maternal mental health and child well-being within tea garden communities in limited resource settings like Bangladesh. To address this gap, the present study adopted a mixed-methods approach to assess the mental health status of mothers working in the tea gardens of the Moulvibazar region in Bangladesh and examined its impact on children’s well-being. The study also aimed to identify the key socio-economic, occupational and contextual factors shaping maternal mental health in this critically underserved population.

## Methods

### Study design and participants

A convergent cross-sectional mixed-methods study employing both quantitative and qualitative approaches was conducted (Katz-Buonincontro and Katz-Buonincontro, 2024). A combination of purposive and stratified random sampling was used, guided by operational feasibility. The four tea gardens were purposely selected from Sreemangal sub-district based on geographic distribution, workforce size, management willingness to facilitate access and an established working relationship with IDEA. We acknowledge that the requirement for management cooperation introduces gatekeeper influence at the garden selection level, and that gardens with more cooperative management may differ systematically from those not included. The combined sampling frame across the four gardens comprised approximately 620 eligible mothers, identified from workforce records and residential lists prepared jointly by garden supervisors and IDEA field coordinators, cross-checked against village council household records. Within each garden, stratified random sampling was applied using employment status (permanent vs. non-permanent) as the primary stratum, with systematic random sampling used within each stratum. Of the 478 mothers initially approached, 451 were included in the final sample; 14 declined participation, 9 were unavailable on the day of data collection and 4 did not meet eligibility criteria.

The quantitative sample size was calculated a priori for prevalence estimation, given the absence of prior population-specific data from tea garden communities. Assuming a conservative prevalence of 50%, a 95% confidence interval, a margin of error of ±5% and a design effect of 1.5 to account for the clustered sampling design across four gardens, the minimum required sample was estimated at 384. Inflating by 15% to account for anticipated non-response and eligibility exclusions yielded a target of approximately 450 participants. The final achieved sample of 451 met this target. It should be noted that the study was not formally powered for subgroup comparisons, and associations reported between maternal mental health and child behavioural outcomes should be interpreted as preliminary and hypothesis generating rather than confirmatory.

Data were collected between 15 and 30 September 2025 from four tea gardens, Rajghat, Patrokhola, Horinganj and Shidurkhan in Moulvibazar District, Bangladesh. In the quantitative component, survey data were collected from 451 working mothers who had at least one child aged between 2 and 7 years. Participants were required to be biological mothers who were currently employed (including both permanent and non-permanent workers) and co-residing with at least one child aged 2–7 years; in cases where mothers had more than one eligible child, information was collected regarding the youngest child. Qualitative data were included to capture nuanced perspectives and contextual factors that quantitative measures alone could not reveal. The qualitative sample size was guided by the principle of data saturation, with interviews and discussions conducted until no new themes or insights emerged from the data. Interview was conducted from 22 to 25 September 2025. The qualitative component comprised 11 key informant interviews (KIIs) with community stakeholders (Table 1). In addition, two focus group discussions (FGDs) were conducted with working mothers from different settings, comprising 11 and 10 participants, respectively (Table 2).

**Table 1.** Characteristics of Key Informant Interviews’ participants of the mental health of working mothers study conducted in Moulvibazar, Bangladesh, 2025 Table 1. long description.

Participant ID	Gender	Tea garden/Organisation name	Occupation	Age (years)	Experience (years)
IF002	Male	Patrokhola Tea Garden	Shardar (Chief)	48	18
IF003	Male	Patrokhola Durga Temple	Temple caretaker	45	20
KM004	Male	Sreemangal, Moulvibazar	Panchayat (village council) President	58	25
KM005	Male	–	Tea Garden manager	46	20
KM006	Male	Horinganj Tea Garden	Head Teacher	39	14
KM007	Male	Non-profit organisation in Sreemangal, Moulvibazar	Job holder	60	32
KM008	Male	Sreemangal, Moulvibazar	Priest	47	17
KM009	Female	Early Childhood Development (ECD)	Teacher	42	6
IAO10	Male	Family Planning Office, Women’s Division	Employee	57	34
IA011	Female	Office of the Deputy Director, Women’s Affairs	Employee	50	24
IA012	Male	Shidurkhan Tea Garden	Medical Dresser	49	3

**Table 2.** Characteristics of Focus Group Discussion A and B participants conducted in two tea gardens of Moulvibazar, 2025 Table 2. long description.

Participants ID	Age (years)	Gender	Marital Status	No. of Children	Occupation	Employment Status	Husband’s Occupation	Experience (years)
A1	40	Female	Widow	1	Picking leaves	Permanent	Labour	3
A2	25		Married	1	Picking leaves	Permanent	Labour	4
A3	25		Married	1	Picking leaves	Permanent	Labour	2
A4	20		Married	1	Picking leaves	Permanent	Labour	2
A5	21		Married	1	Picking leaves	Permanent	Labour	3
A6	28		Married	2	Picking leaves	Permanent	Labour	6
A7	25		Married	1	Picking leaves	Permanent	Labour	2
A8	36		Married	1	Picking leaves	Permanent	Unemployed	12
A9	24		Married	1	Picking leaves	Permanent	Labour	2
A10	35		Widow	1	Picking leaves	Permanent	Labour	7
A11	23		Married	2	Picking leaves	Permanent	Car driver	6
B1	25		Married	1	Picking leaves	Permanent	Unemployed	2
B2	24		Married	1	Picking leaves	Permanent	Labour	2
B3	24		Married	2	Picking leaves	Permanent	Labour	2
B4	24		Married	1	Picking leaves	Permanent	Unemployed	1
B5	25		Married	1	Picking leaves	Permanent	Labour	3
B6	24		Married	1	Picking leaves	Permanent	Labour	4
B7	38		Married	1	Picking leaves	Permanent	Unemployed	10
B8	25		Married	1	Picking leaves	Permanent	Labour	4
B9	27		Married	3	Picking leaves	Permanent	Unemployed	15
B10	35		Married	3	Picking leaves	Permanent	Labour	3

KII participants were purposively selected to represent five institutional domains relevant to the structural determinants of maternal mental health in tea garden communities: workplace governance (garden management and supervisory staff); community governance (village council leadership); healthcare (medical dresser); civil society and NGO sector (NGO programme officer) and government (Women’s Affairs and Family Planning officers). A religious leader and a temple caretaker were included given their recognised roles as trusted informal sources of community psychosocial support in South Asian rural contexts. A school head teacher was included to provide perspective on children’s educational well-being. Access to garden management participants was limited by the sensitivity of discussions concerning working conditions; only one management-level participant was available across the four gardens. The single healthcare informant, the most senior healthcare worker resident in the study gardens during the data collection period, provides a valuable frontline perspective but limits the depth of clinical insight available from the qualitative component. These constraints are acknowledged as limitations of the KII sample.

### Data collection tools

#### Quantitative tools

1. The Bangla version of the Depression, Anxiety, Stress Scale-21 (DASS-21) to assess maternal psychological distress (Ahmed et al., 2022).
2. The Bangla parent reported version of the Strengths and Difficulties Questionnaire (SDQ) to assess child behavioural and emotional well-being (Mullick and Goodman, 2001).
3. The Bangla version of the World Health Organisation–Five Well-Being Index (WHO-5) to measure subjective well-being (Faruk et al., 2021a,b; Rahaman et al., 2025).

In the present sample (*n* = 451), internal consistency was acceptable for both the DASS-21 (Cronbach’s α = 0.78) and the WHO-5 (Cronbach’s α = 0.77). Internal consistency of the SDQ total difficulties score was not calculated given its composite multidimensional structure; subscale-level psychometric properties in a Bangla-speaking population are reported in Mullick and Goodman (2001).

The Bangla version of the DASS-21 and WHO-5 were used in this study and the SDQ was administered in Bangla during the interview process. These tools had previously been used and validated in Bangla-speaking population. Although no cognitive testing was conducted separately for the tea garden population, trained interviewers restated the questions when necessary and explained them in simple language so that the participants could understand them properly.

#### Qualitative tools

Qualitative tools included semi-structured interview and FGD guides developed through literature review, contextual understanding of tea garden communities and expert consultation in mental health and public health research. The interview and discussion guides, including prompts, were developed by the authors and pilot-tested with two individuals prior to the commencement of the study to ensure clarity and relevance.

### Data collection process

Many participants had low level of formal education. Therefore, the quantitative data were administered through face-to-face interviews rather than self-completion. The interviews were conducted in Bangla and where necessary, questions were explained in simple language so that participants with low literacy could understand.

The interviews and FGDs were conducted by the first two authors of the study. The first author was a male research consultant, holds an MSc in Clinical Psychology, and has approximately 3 years of qualitative research experience and training. The second author is a female, working as a research assistant and holds a BSc in Microbiology and Immunology. She has completed two qualitative research training programmes and has approximately 2 years of qualitative research experience. No prior relationship was established with participants before data collection. However, brief rapport-building was conducted immediately prior to the interviews and discussions to facilitate open communication. Participants were informed that the researchers were part of the research team; however, they were not informed about the interviewers’ roles as study authors. The interviewers were trained in qualitative research methods and maintained a neutral and non-judgmental approach during data collection. Efforts were made to minimise bias by following structured guides and allowing participants to freely express their views without influence from the researchers’ personal assumptions or expectations.

KIIs were conducted at the participants’ respective workplaces. For the two FGDs, a separate room was arranged in advance to ensure a suitable and private setting. Only the participants, interviewers, and note-takers were present during the sessions. No repeat interviews were conducted, and transcripts were not returned to participants for review or comments. Two eligible participants declined to participate due to work schedule constraints.

### Data analysis

#### Quantitative data

Quantitative data were analysed using IBM SPSS Statistics, version 26. Variables were classified as follows: continuous variables included age (years), monthly income (BDT), daily working hours, days worked per week, number of children and total scores on the DASS-21 subscales, WHO-5 and SDQ; categorical variables included educational qualification, marital status, employment type, occupation, childcare arrangement during working hours and income group.

Descriptive statistics were used to characterise the sample. Continuous variables are presented as means with standard deviations (SD); categorical variables are presented as frequencies and percentages.

For inferential analysis, the choice of test was determined by variable type and distribution. The Shapiro–Wilk test was used to assess normality for all continuous outcome variables. Where normality assumptions were met, independent samples t-tests were used to compare mean SDQ total difficulty scores between mothers with and without depression, specifically to examine the association between maternal depression and child behavioural difficulties (reported in Table 5 and the association analysis). Where group comparisons involved more than two categories, for example, comparing DASS-21 and WHO-5 scores across age groups, educational qualification, income category, employment type and childcare arrangement, one-way ANOVA was used where normality assumptions were satisfied. Associations between categorical variables were examined using chi-square tests of independence.

Pearson correlation coefficients were calculated to examine relationships between continuous variables where normality was confirmed. All tests were two-tailed. A significance threshold of *p* < 0.05 was applied throughout.

We acknowledge that the analysis involved multiple comparisons across several socio-demographic and occupational variables simultaneously, which increases the risk of Type I error. Corrections for multiple comparisons (such as Bonferroni adjustment) were not applied, consistent with the exploratory and descriptive intent of these analyses. However, findings from subgroup comparisons should be interpreted with appropriate caution, and statistically significant associations reported in this study should be regarded as hypothesis generating rather than confirmatory. Future studies with pre-specified primary hypotheses and appropriate correction procedures are needed to confirm these findings.

1. For the DASS21 calculation, the Likert scale was scored from 0 to 3, where it did not apply to me, marked as 0, and 3 indicated “applied to me very much or most of the time.” No items were reverse-coded. Since the version used in this study was a shorter form of the DAS scale, the initial total score was multiplied by 2 to obtain the final score. Based on the final scores depression was classified as normal (0–9), mild (10–13), moderate (14–20), severe (21–27) and extremely severe (28+). Additionally, anxiety was classified as normal (0–7), mild (8–9), moderate (10–14), severe (15–19) and extremely severe (20+), while stress as normal (0–140, mild (15–18), moderate (19–25), severe (26–33) and extremely severe (34+). (Ahmed et al., 2022). For the Depression Anxiety Stress Scales, participants were grouped into categories for analytical convenience. In this study, “high levels” refer to those scoring in the severe and extremely severe ranges combined, while “vulnerable” refers to those in the mild to moderate ranges. Participants classified within the moderate category were considered a “vulnerable” group, as they may be at increased risk of progressing to more severe levels of depression, anxiety and stress. These groupings were created to facilitate interpretation and comparison and do not represent formal diagnostic classifications.
2. For the WHO-5 Well-being Index, the Likert scale was scored from 0 to 5, with five assigned for “All the time” and 0 for “At no time.” The cut-off scores for the three categories were 0–13 for poor well-being, 14–19 for moderate and above 20 for good well-being (Rahaman et al., 2025). For the WHO-5 Well-Being Index, categorised levels of well-being (poor, moderate and good) were used for interpretive purposes based on selected cut-off values. The term “vulnerable to poor psychological well-being” was used to describe individuals in the moderate category. These categorisations were applied to aid analysis and should not be interpreted as clinical thresholds.
3. For the SDQ scale, “Not true” was scored 0, while “Certainly true” was scored 2. SDQ questions 6, 7, 14 and 21 were reverse-coded. Overall strength and difficulties were calculated, with cut-off values of 0–15 regarded as normal, 16–19 as borderline and 20–40 as abnormal (Mullick and Goodman, 2001).

Of the 451 participants included in the final sample, item-level missing data were minimal. The DASS-21 and WHO-5 were fully complete for all 451 respondents. For the SDQ, three respondents (0.7%) had one missing item each in the Peer Problems subscale; these were addressed using mean imputation at the subscale level, consistent with established SDQ scoring guidance (Goodman, 1997). For socio-demographic variables, nine respondents (2.0%) did not report monthly income; these cases were retained in all analyses not involving income and excluded from income-specific comparisons only. No case-level or multiple imputation was performed. All primary analyses were conducted on complete cases for each relevant variable.

#### Qualitative data

Qualitative data from all KIIs and FGDs were audio-recorded with participant consent, transcribed verbatim in Bangla, and translated into English by the field researchers. A primarily inductive coding approach was adopted, consistent with the exploratory nature of the study and the limited prior literature on this population, although the research questions provided a loose sensitising framework that guided the initial organisation of codes into broad thematic domains, an approach characterised as hybrid thematic analysis (Fereday and Muir-Cochrane, 2006).

An initial codebook was generated following the first two KII transcripts, comprising 34 preliminary codes organised across five broad domains. The codebook was shared with the analytic team and revised through discussion before application to subsequent transcripts. New codes were added as they emerged and existing codes were refined, merged or split as the analysis progressed. The finalised codebook comprised 58 codes organised across six thematic domains.

To assess analytic consistency, approximately 20% of transcripts (two KII and one FGD transcript) were independently coded by a second researcher using the finalised codebook. Inter-rater disagreements were identified in 11% of coded segments and resolved through structured discussion between the two coders until consensus was reached. No cases required third-party adjudication. The remaining transcripts were coded by the lead researcher using the consensus-revised codebook.

Reflexivity was maintained through analytic journaling by the lead investigator, regular team debriefs at key points during data collection and analysis, and critical discussion of emerging interpretations with team members holding diverse disciplinary and lived-experience perspectives. Credibility was strengthened through methodological triangulation, themes from KII and FGD data were developed independently before integration, and through the use of thick descriptive data, including participant quotations selected to represent the breadth of perspectives captured. The finalised codebook is available from the corresponding author on reasonable request.

### Ethical consideration

Ethical approval was taken from the Ethical Review Board of the Public Health Foundation, Bangladesh (PHFBD-ERC-FP-R-38/2025). Written informed consent was taken from the participants before the interviews were conducted. Participants were informed of the study’s purpose, the voluntary nature of participation, their right to withdraw at any stage and their right to skip questions they preferred not to answer.

## Results

### Quantitative findings

A total of 451 participants were included in the study. The mean age of the participants was 29.24 ± 5.04 years. Most participants were aged 25–34 years (*n* = 330, 73.2%), followed by 35–44 years (*n* = 62, 13.7%), 18–24 years (*n* = 53, 11.8l) and 45–54 years (*n* = 6, 1.3%). Regarding educational qualification, 39.7% (*n* = 179) had completed primary education, 25.3% (*n* = 114) had no formal education, 21.5% (*n* = 97) had secondary education and 13.5% (*n* = 61) had completed SSC/HSC or higher. The majority of participants were married (*n* = 437, 96.9%), while 2.0% (*n* = 9) were widowed and 1.1% (*n* = 5) were separated. The mean number of children was 1.9 ± 0.89, with 41.9% (*n* = 189) having two children, 37.5% (*n* = 169) having one child, 15.1% (*n* = 68) having three children and 5.6% (*n* = 25) having more than three children.

In terms of occupation, the majority were involved in leaf picking (*n* = 332, 73.6%), followed by temporary workers (*n* = 54, 12.0%), garden workers (*n* = 38, 8.4%), others (*n* = 16, 3.5%) and factory workers (*n* = 11, 2.4%). The mean daily working hours were 8.03 ± 0.58 h, and participants worked an average of 6.09 ± 0.55 days per week. The mean monthly income was 8,701.4 ± 3,716.3 BDT. Most participants fell into the low-income category (<12,500 BDT; *n* = 379, 84.0%), while 15.1% (*n* = 68) were in the middle-income group (12,500–21,000 BDT) and only 0.7% (*n* = 3) belonged to the upper-income group (>21,000 BDT) (Table 3) (Fidah et al., 2023).

**Table 3.** Demographic characteristics of the working mothers in Moulvibazar tea gardens, Bangladesh, Bangladesh Table 3. long description.

Variables	Categories	Number (N)	Percentage (%)	Mean ± SD
Age (years)				29.24 ± 5.04
	18–24	53	11.8	
	25–34	330	73.2	
	35–44	62	13.7	
	45–54	6	1.3	
Educational qualification				
	None	114	25.3	
	Primary	179	39.7	
	Secondary	97	21.5	
	SSC/HSC or more	61	13.5	
Marital status				
	Married	437	96.9	
	Separated	5	1.1	
	Widow	9	2.0	
Family type				
	Nuclear	283	62.7	
	Joint	168	37.3	
Number of children				1.9 ± 0.89
	One child	169	37.5	
	Two children	189	41.9	
	Three children	68	15.1	
	More than three children	25	5.6	
Occupation				
	Picking leaves	332	73.6	
	Factory workers	11	2.4	
	Temporary workers	54	12	
	Garden workers	38	8.4	
	Others	16	3.5	
Work status				
	Permanent	158	35	
	Non-permanent	293	65	
Work hours				8.03 ± 0.58
Work days				6.09 ± 0.548
Income				8,701.4 ± 3,716.3
	Low-income (<12,500 BDT)	380	84	
	Middle-income (12,500–21,000)	68	15.1	
	Upper income (>21,000)	3	0.7	

Table 4 presents the psychological well-being status of working mothers in the Moulvibazar tea gardens. About 5.8% (*n* = 26), 20.6% (*n* = 93) and 16.4% (*n* = 74) working mothers have high levels of depression, anxiety and stress, respectively. Additionally, 32.4% (*n* = 146), 29.9% (*n* = 135) and 31.7% (*n* = 143) working mothers are vulnerable to developing high-level depression, anxiety and stress, respectively. In terms of psychological well-being, 33.9% (*n* = 153) working mothers have poor well-being and 41% (*n* = 185) working mothers are vulnerable to poor psychological well-being.

**Table 4.** Psychological well-being assessment of working mothers in Moulvibazar tea gardens Table 4. long description.

Descriptive statistics	Categories	Number (N)	Percentage (%)	Mean ± SD
Depression level				11 ± 6.2
	Normal	170	37.7	
	Mild	109	24.2	
	Moderate	146	32.4	
	Severe	21	4.7	
	Extremely severe	5	1.1	
Anxiety level				9.3 ± 6.3
	Normal	179	39.7	
	Mild	44	9.8	
	Moderate	135	29.9	
	Severe	60	13.3	
	Extremely severe	33	7.3	
Stress level				18 ± 7.5
	Normal	152	33.7	
	Mild	81	18	
	Moderate	144	31.9	
	Severe	62	13.7	
	Extremely severe	12	2.7	
Well-being				15.39 ± 7.5
	Poor	153	33.9	
	Moderate	185	41	
	Good	113	25.1	

Table 5 shows the distribution of depression, anxiety, stress and well-being levels among working mothers in the Moulvibazar tea gardens according to selected demographic characteristics. Age was significantly associated with stress level (*p* = 0.014) and overall well-being (*p* = 0.002). Higher proportions of moderate and severe stress, as well as poor well-being, were observed among mothers aged 25–34 years compared with other age groups. No statistically significant association was found between age and depression (*p* = 0.124) or anxiety (*p* = 0.298). Educational qualification showed a significant association with depression (*p* < 0.001) and well-being (*p* < 0.001). Participants with no formal education accounted for a higher proportion of severe and extremely severe depression and poor well-being. However, education was not significantly associated with anxiety (*p* = 0.30) or stress (*p* = 0.932). Marital status was significantly associated with depression (*p* = 0.006) and anxiety (*p* = 0.022). Married participants constituted the majority across all severity categories, including severe and extremely severe outcomes. No significant associations were observed between marital status and stress (*p* = 0.807) or well-being (*p* = 0.169). No statistically significant association was found between childcare arrangement and anxiety levels (*p* = 0.171). Non-permanent workers mostly faced severe to extremely severe anxiety. Additionally, an extreme level of stress was also observed among the low-income group, and when children are left with family members. Age, educational qualification, income and caring for children during work were statistically significant factors affecting the well-being index of mothers working in the tea garden community in Moulvibazar. Participants in the 25–34 age group, those with primary education, married participants and those engaged in leaf picking mostly exhibited poor well-being (Table 5). It is important to note that the observed higher proportions of married participants, individuals aged 25–34, leaf pickers, and mothers with two children across severity categories should be interpreted in light of the overall sample composition, as these groups were overrepresented in the sample and therefore do not necessarily indicate associations with distress levels.

**Table 5.** Level of depression, anxiety and stress of mothers working in Moulvibazar tea gardens based on demographic factors Table 5. long description.

Variables	Depression level				Anxiety level				Stress level				Well-being	
	Moderate (*n* = 146)	Severe (*n* = 21)	Extremely severe (*n* = 5)	P-value	Moderate (*n* = 135)	Severe (*n* = 60)	Extremely severe (*n* = 33)	P-value	Moderate (*n* = 144)	Severe (*n* = 62)	Extremely severe (*n* = 12)	P-value	Poor	P-value
**Age (years)**				0.124				0.298				**0.014**		**0.002**
18–24	11 (7.5%)	2 (9.5%)	1 (20%)		16 (11.9%)	6 (10.0%)	4 (12.1%)		13 (9.0%)	5 (8.1%)	–		12 (7.8%)	
25–34	120 (82.2%)	14 (66.7%)	4 (80%)		102 (75.6%)	44 (73.3%)	23 (69.7%)		109 (75.7%)	41 (66.1%)	10 (83.3%)		118 (77.1%)	
35–44	15 (10.3%)	5 (23.8%)	–		17 (12.6%)	10 (16.7%)	6 (18.2%)		21 (14.6%)	14 (22.6%)	2 (16.7%)		22 (14.4%)	
45–54	–	–	–		–	–	–		1 (0.7%)	2 (3.2%)	–		1 (0.7%)	
**Education qualification**				**<0.001**				0.30				0.932		**<0.001**
None	48 (32.9%)	15 (71.4%)	2 (40%)		34 (25.2%)	19 (31.7%)	12 (36.4%)		39 (27.1%)	14 (22.6%)	3 (25.0%)		22 (14.4%)	
Primary	6.2 (42.5%)	2 (9.5%)	1 (20%)		48 (35.6%)	24 (40.0%)	12 (36.4%)		63 (43.8%)	21 (33.9%)	5 (41.7%)		72 (47.1%)	
Secondary	27 (19%)	4 (19%)	1 (20%)		30 (22.2%)	11 (18.3%)	4 (12.1%)		26 (18.1%)	17 (27.4%)	4 (33.3%)		45 (29.4%)	
SSC/HSC or more	9 (6.2%)	–	1 (20%)		23 (17.0%)	6 (10.0%)	5 (15.2%)		16 (11.1%)	10 (16.1%)	–		14 (9.2%)	
**Marital status**				**0.006**				**0.022**				0.807		0.169
Married	141 (96.6%)	18 (85.7%)	5 (100%)		134 (99.3%)	57 (95.0%)	29 (87.9%)		137 (95.1%)	61 (98.4%)	12 (100.0%)		146 (95.4%)	
Separated	1 (0.7%)	–	–		–	–	1 (3.0%)		1 (0.7%)	1 (1.6%)	–		2 91.3%)	
Widow	4 (2.7%)	3 (14.3%)	–		1 (0.7%)	3 (5.0%)	3 (9.1%)		6 (4.2%)	–	–		5 (3.3%)	
**Number of children**				0.126				**0.036**				0.155		0.641
One	58 (39.7%)	7 (33.3%)	2 (40%)		52 (38.5%)	32 (53.3%)	14 (42.4%)		58 (40.3%)	15 (24.2%)	1 (8.3%)		56 (36.6%)	
Two	61 (41.8%)	10 (47.6%)	3 (60%)		57 (42.2%)	19 (31.7%)	16 (48.5%)		62 (43.1%)	27 (43.5%)	9 (75%)		67 (43.8%)	
Three	17 (11.6%)	3 (14.3%)	–		18 (13.3%)	7 (11.7%)	2 (6.1%)		16 (11.1%)	15 (24.2%)	2 (16.7%)		24 (15.7%)	
More than three	10 (6.8%)	1 (4.8%)	–		8 (5.9%)	2 (3.3%)	1 (3%)		8 (5.6%)	5 (8.1%)	–		6 (3.9%)	
**Occupation**				**<0.001**				**0.003**				**<0.001**		0.227
Picking leaves	114 (78.1%)	17 (81%)	4 (80%)		85 (63.0%)	42 (70.0%)	23 (69.7%)		109 (75.7%)	34 (54.8%)	7 (58.3%)		107 (69.9%)	
Factory workers	4 (2.7%)	2 (9.5%)	–		3 (2.2%)	–	4 (12.1%)		3 (2.1%)	2 (3.2%)	–		3 (2.0%)	
Temporary workers	19 (13%)	2 (9.5%)	1 (20%)		19 (14.1%)	8 (13.3%)	3 (9.1%)		15 (10.4%)	8 (12.9%)	2 (16.7%)		25 (16.3%)	
Garden workers	2 (1.4%)	–	–		24 (17.8%)	7 (11.7%)	–		15 (10.4%)	17 (27.4%)	3 (25.0%)		13 (8.5%)	
Others	7 (4.8%)	–	–		4 (3.0%)	3 (5.0%)	3 (9.1%)		2 (1.4%)	1 (1.6%)	–		5 (3.3%)	
**Income**				0.271				**<0.001**				0.838		**0.001**
Low-income (<12,500 BDT)	119 (81.5%)	19 (90.5%)	5 (100%)		124 (91.9%)	57 (95%)	30 (90.9%)		121 (84.0%)	54 (87.1%)	11 (91.7%)		143 (93.5%)	
Middle-income (12,500–21,000)	27 (18.5%)	2 (9.5%)	–		9 (6.7%)	3 (5%)	3 (9.1%)		21 (14.6%)	8 (12.9%)	1 (8.3%)		9 (5.9%)	
Upper income (>21,000)	–	–	–		2 (1.5%)	–	–		2 (1.4%)	–	–		1 (0.7%)	
**Carer for children during work**				**<0.001**				0.171				**<0.001**		**0.026**
Family members	102 (69.9%)	16 (76.2%)	5 (100%)		62 (45.9%)	40 (66.7%)	27 (81.8%)		95 (66.0%)	24 (38.7%)	8 (66.7%)		89 (58.2%)	
Neighbours	18 (12.3%)	1 (4.8%)	–		20 (14.8%)	5 (8.3%)	0 (0.0%)		14 (9.7%)	6 (9.7%)	–		21 (13.7%)	
Daycare	22 (15.1%)	2 (9.5%)	–		50 (37.0%)	13 (21.7%)	4 (12.1%)		30 (20.8%)	31 (50.0%)	4 (33.3%)		38 (24.8%)	
Take their children to work	4 (2.7%)	2 (9.5%)	–		3 (2.2%)	2 (3.3%)	2 (6.1%)		5 (3.5%)	1 (1.6%)	–		5 (3.3%)	

Assessment using the SDQ indicated that a high proportion of children fell within the abnormal range (87.8%, *n* = 396), while 9.1% (*n* = 41) were classified as borderline and only 3.1% (*n* = 14) as normal. A notably high proportion of children (87.8%, *n* = 396) were classified as having abnormal scores on the parent-reported SDQ, which should be interpreted with caution given the reliance on maternal reporting and the potential influence of reporting bias. Furthermore, an independent samples t-test was conducted to examine the association between maternal depression and child behavioural difficulties, as measured by the SDQ total difficulty score. Maternal depression was operationalised as a DASS-21 depression subscale score ≥ 10 (mild to extremely severe range), with mothers scoring in the normal range (0–9) as the comparison group. Children of mothers with depression (*n* = 172, mean SDQ = 24.83, SD = 4.21) showed significantly greater total difficulty scores than children of mothers without depression (*n* = 279, mean SDQ = 22.96, SD = 4.67); *t*(449) = 4.11, *p* = 0.011, Cohen’s *d* = 0.42. Both group means fall within the abnormal SDQ range (≥20), indicating that child behavioural difficulties were elevated across the full sample regardless of maternal depression status. The magnitude of the between-group difference should therefore be interpreted in this context, and the finding treated as preliminary given the cross-sectional design and reliance on maternal report for both variables.

### Qualitative findings

#### Participant’s characteristics

The six themes identified across KIIs and FGDs do not function as independent findings but constitute a coherent structural narrative. Themes 1–4 represent interacting dimensions of cumulative structural risk, poor mental health literacy, excessive workload, unsafe conditions and financial deprivation that together constitute the environment in which maternal distress is produced and sustained. Theme 5 identifies the mediating conditions that either compound this risk (restricted autonomy and absent institutional support) or partially buffer it (supportive management, village councils and NGO presence). Theme 6 maps participant-identified solutions directly onto these protective factors, suggesting that effective intervention need not be constructed from outside the community but rather built upon what participants themselves identify as already working. Themes 2 and 4 were dominant across all participant types and constituted the strongest points of convergence between KII and FGD data. The most notable divergence between data sources concerned the characterisation of management practices and the gap between institutional awareness of mental health and its community-level internalisation, both of which are discussed following the thematic findings.

Unless otherwise stated, qualitative findings are presented as participant-reported perceptions rather than verified community realities. Where accounts were consistent across multiple participant types and data sources, this convergence is noted as strengthening the credibility of the finding. Where accounts were contested, divergent or limited to a single informant, this is explicitly contextualised. Claims corroborated by the quantitative data are cross-referenced to the relevant quantitative findings.

#### Theme 1: Mental health awareness and perceptions

This theme captures the current state of mothers’ mental health, including perceptions, prevalence and indicators of psychological distress. Participants reported being able to recognise poor mental health through facial expressions and behaviours such as lack of focus, anxiety due to poverty or nutrition and violent moods.

The symptoms of mental problems are that she will be in a bad mood. Just by looking at her, you can understand that she is worried. Maybe it seems that I want to talk to her, but she does not want to talk, if you ask her something, she may answer it but will not show much interest. She will misbehave with her neighbour or her child, or her husband. Even if she is told good things, it is seen that she is taking it negatively

(KM006, KII).

However, the community often misinterprets these indicators as ego or normal stress rather than a mental health concern. Participants offered divergent accounts of whether mental health awareness in the community was increasing or improving. Some KII informants, particularly those in NGO and government roles, perceived a gradual improvement attributable to awareness programmes. Others, including community leaders and working mothers in both FGDs, reported that mental health problems remained largely unrecognised and normalised, with distress accepted as an inevitable condition of life. These divergent perspectives are treated here as reflective of participants’ different institutional positions and levels of proximity to formal awareness programming, rather than as evidence for or against any objective trend in community awareness.

I can’t say whether it’s increasing or decreasing. But as I said, they don’t recognise mental health problems. They just live with those issues, believing that this is normal. They think, ‘My mother lived this way, my mother-in-law lived this way, I’m living this way.’ What seems like a problem to us feels normal to them

(IA011, KII).

#### Theme 2: Workload, household burden and domestic support

This theme combines the pressures of workload, exhaustion and the lack of domestic support. Mothers work long hours in tea gardens, often 6–7 days a week, while also managing household chores and childcare, leaving them with little opportunity to rest.

A mother’s health will be alright. Just taking medicine and staying at home will not make health right. Some rest is needed. Now the mother doesn’t have this time. Now she has to go to duty within one hour of waking up in the morning. After returning from duty, she has to bathe and then immediately give attention to the children; then she has to enter the kitchen again. It will be 10 to 11 at night while cooking. She eats herself and feeds the children. After that, there are the house chores, this and that, all the fuss until 1 AM! When does the mother rest! And when will the mother’s health be good from rest!

(KM008, KII).

Domestic support, including help from husbands, family members or neighbours, significantly impacts mothers’ well-being. The lack of support increases stress, while active support reduces their burden.

Many of the husbands take no responsibility; they don’t contribute to family expenses and spend their earnings on themselves. This causes constant tension in families. But not all are bad, there are good families too. Some husbands are caring; they look after their children’s education, and you can see the difference, those women look cheerful, happy, and content. However, many of them marry at a very young age, and because of that, their health is often already compromised

(IA10, KII).

#### Theme 3: Adverse working conditions and safety risks

Whereas Theme 2 addressed the compounded burden of domestic and caregiving responsibilities, this theme focuses on the physical, regulatory and safety conditions within the tea gardens themselves as a distinct and additional source of maternal distress. Mothers often work in harsh weather, under physical hazards such as lightning, snakes or falling trees and face exploitative practices including delayed or partial payment. Attendance tracking via biometric systems adds further stress, especially when minor errors result in loss of wages.

They can’t collect the required quota of leaves on time. The work (meeting the quota) is stressful because the section is difficult (overgrown), there’s a jungle, sun, sweat. You’ll see, when they go to work, there’s no vehicle; if a vehicle comes, there’s no Babu (supervisor); if the Babu comes, the circular says two kgs of leaves on paper, but six kgs are required for plucking. Not everyone knows how to read and write. No one can track it

(IF003, KII).

Working mothers in both FGDs and lower-level KII informants, including the Sardar and the medical dresser, consistently described the daily leaf-picking quota as a source of significant psychological stress, reporting that actual quota demands exceeded formally stated targets and that shortfalls resulted in wage penalties. These accounts were consistent across FGD participants from two different gardens and were corroborated by the supervisory-level KII informant, who attributed the pressure to company targets rather than local management discretion. Management-level perspectives on the quota system were limited to a single KII participant and should not be taken as representative of garden management as a whole. Quota-related distress is therefore reported as a consistently held perception among workers and front-line staff rather than as a verified organisational practice. Mothers sometimes continue working during late pregnancy or illness due to financial pressure, while safety and rest provisions are inadequate.

Even after working a whole week, we don’t get full attendance wages. They give us two or three days’ worth and say, ‘Take this, more will be given later.’ This way, they just keep making us work by giving false assurance

(B2, FGD).

#### Theme 4: Socio-economic barriers and basic needs deprivation

The socio-economic conditions described here operate at a broader structural level than the occupational experiences in Theme 3, shaping the material constraints within which both domestic and workplace pressures are experienced and limiting women’s capacity to seek relief from either. Financial constraints heavily affect the mental health of mothers and their families. Despite long hours, wages are insufficient to meet household needs. Loan schemes, garden closures and inflation exacerbate the financial stress. Mothers often struggle to provide nutrition, education and basic healthcare for their children.

Just after a whole year, we got only six-thousand-taka bonus. Now I think, should I spend it for myself, or for my daughters, or for weddings, or for food and clothes during the festival? I face all kinds of worries, money worries, household worries, and mental stress. Mothers especially have to bear all of this. That’s why they naturally feel unhappy

(IF02, KII).

Inadequate healthcare, sanitation and access to clean water further compound stress. Free services exist, but participants reported poor quality and insufficient resources.

We don’t have an ambulance in the garden. No ambulance. Suppose a pregnant woman urgently needs to be taken outside. Our medical unit here is big, twenty to thirty people are employed there, they’re eating salaries (taking salaries), but in reality, nothing is there. They don’t even have a proper syringe to push an injection. That’s what I feel. If those things existed, then the doctors could handle many cases themselves. But they can’t do everything by hand. They need proper equipment. And even that basic equipment is not here. The company doesn’t provide it. Nothing

(KM008, KII).

#### Theme 5: Vulnerability, helplessness and protective factors

Women often experience helplessness due to lack of decision-making power, restricted mobility and minimal institutional support. Barriers can arise from family, community and management, leaving mothers with little recourse to improve their situation.

The barriers mostly come from within the family. First of all, if she says, ‘I want to go seek help for this,’ her husband won’t allow her to go. He’ll say, ‘You don’t need to go, you’re fine,’ or ‘If you go, I’ll leave you. I won’t keep you. I’ll throw you and my child out.’ So that woman won’t go. Even if she’s being abused and wants to seek justice, they’ll threaten her…

(IA011, KII).

Despite these vulnerabilities, some protective factors exist. Supportive managers, local council committees, NGOs and neighbours can alleviate stress and provide critical assistance, including emergency support, monetary help and advocacy.

Some managers are a little understanding, especially those from poorer backgrounds. But the newer ones that come, they often don’t understand. For example, we had one manager before, he was kind, treated people well, and thought about workers’ welfare. But because of that, he lost his job. Now we have a new manager, if you’re not at work by 8 a.m., he’s already standing there, forcing people to come quickly. But managers are not all the same, they change every few months. Some are good and understanding, some are not at all. As for staff and sardars (supervisors), they try to behave well with workers, but because of company pressure, they can only do so much

(IF002, KII).

#### Theme 6: Recommendations for improvement

Participants emphasised solutions to improve mental health and well-being, including accessible childcare, improved healthcare, sanitation and proper work schedules. Education and awareness programmes are also crucial for both mothers and children. NGOs and government initiatives should expand to provide more consistent, long-term support.

NGOs are actually working! However, their work is becoming limited, yes, limited. Many NGOs are not even there anymore. Donors are reducing support. So, I think NGOs should take on more work in tea gardens, such as in health, mental health, menstrual hygiene, and basic hygiene. In these areas, they should undertake more work. Nutrition too. These are the areas they should focus on, as they will have a significant impact on their lives

(KM007, KII).

One KII informant, a programme officer at a non-profit organisation in Sreemangal, described a reduction in NGO activity in tea garden communities, attributing this to declining donor funding. This perspective was not corroborated by other KII informants and is therefore reported as a single stakeholder’s account rather than an established pattern. It is included because it was raised as a concern relevant to the sustainability of identified protective factors, not because it reflects a verified sectoral trend.

The owners could do some things, for example, assign dedicated healthcare staff for women’s health, set up a separate medical section for workers, build proper toilets in the tea gardens, and establish fixed work schedules. Like, a mother could come in the morning, leave at noon to check on her child, and return in the afternoon. The company could arrange that.

(IA012, KII).

## Discussions

This study reveals a substantial burden of depression, anxiety, stress and poor psychological well-being among mothers working in tea garden communities. The findings align with global evidence indicating that women in low-wage, labour-intensive occupations experience elevated mental health risks due to overlapping economic, occupational and social pressures.

### Household support and compounded maternal workload

A key finding of the study is that workload at both the workplace and home negatively affects mothers’ psychological well-being. Given that the sample was predominantly composed of married participants, no meaningful conclusions could be drawn regarding the association between marital status and psychological outcomes. The burden could be due to increased workload after marriage, such as women having to work in the garden, manage household chores and support their husbands, which has been identified in our qualitative findings. According to a study, spouses serve as the first line of defence during the development of any physical or mental illness. The emotional support provided by a spouse positively impacts mental (Choi and Ha, 2011; Reczek et al., 2020). However, in our qualitative findings, many participants shared that husbands often do not help with housework, financial support or childcare and some abandon their wives and children to remarry, which can increase the women’s mental strain. A longitudinal study indicates that dissatisfaction among married women regarding their husbands’ household contributions can harm their mental health (Baek et al., 2024). Conversely, women living in joint families tend to have better mental well-being compared to those in nuclear families. Women in joint families often receive more help with household tasks and childcare, as well as substantial financial support, especially when multiple family members work and contribute to expenses (Zhou et al., 2022; Simanjuntak et al., 2024).

### Chronic financial strain and exploitative working conditions

The primary barrier to the well-being of working mothers is financial hardship. A 2021 study reports that tea company owners in Bangladesh earn about TK14,000 million, while their expenditures on workers’ wages and other expenses (fuel, machinery, maintenance, management staff, etc.) amount to about TK6,000 million (Rahman et al., 2021). Tea garden workers earn approximately TK170 per day after an 8-h shift, although some gardens reported wages set at TK187 per day during our study (Shoaib-Bin-Habib et al., 2025; Al-Amin and Islam, 2020). During the FGDs, working mothers expressed that with this amount, managing household chores and providing proper care for their children is difficult. Consequently, women often work 7 days a week to earn extra income. However, many face exploitation or do not receive full payment for their work. Yet, they continue working due to limited job opportunities and skills, amidst a large population in the tea community. Our quantitative data shows that 73.5% of mothers with high levels of depression, anxiety and stress work as leaf pickers, averaging 6 days of work at 8 hours per day. And since wages are paid daily, women work under direct heat, rain or storms because of a lack of shelters to meet daily quotas. Working in such rough conditions, yet they are exposed to exploitation, such as plucking more tea leaves than allowed without extra pay, or not receiving full weekly wages despite working full weeks. During the FGDs, women also highlighted that the absence of toilets and tubewells causes significant mental stress, as they must relieve themselves in open spaces. After working in extreme heat while standing, they are unable to quench their thirst with the two water bottles they carry from home. Women also have to work during menstruation, with only two sick leave days annually. Studies suggest that 9%–17% of women experience irritability or anxiety during menstruation, which can affect their work performance and mental well-being (Alemu et al, 2025; Hennegan et al., 2022; Ara et al., 2024). In addition to household duties, the tough working environment profoundly impacts their mental health (WHO, 2024).

### Association between the mother’s and the child’s psychological well-being

Mothers who keep their children with family members, when they are at work, mostly have severe or extremely severe depression, anxiety and stress levels. According to a study, for working mothers, children’s well-being during their absence is their major concern (Mansoureh Ahmadifaraz et al., 2013). During the FGDs, many mothers expressed that they feel relieved when their children are at school or day care, as they are not neglected there. In our study, many of the tea gardens did not have a day care. Some of the gardens that did have a daycare facility were only open from 9 am to 11:30 am, after which the children were either put under the responsibility of the family members or neighbours.

Furthermore, studies also suggest that the more children, the poorer the level of mental health has been observed among mothers compared to mothers with more than 3 (Pearson et al., 2019). This justifies why in our study, the level of anxiety has a significant difference across the number of children groups. However, in our study, the majority (41.9%) of mothers had two children, followed by one child (37.5%). And the majority of the mothers with extremely severe depression, anxiety and stress had two children. A possible explanation for this result was identified in our qualitative findings, where participants shared that in the tea garden community, working mothers receive various types of maternal benefits, such as 4 months of paid leave, ration and healthcare services; however, these maternal benefits cease during their third pregnancy. Which is why it can be observed that the majority of the working mothers in our study had one or two children.

While concerns regarding children significantly impact the mental well-being of the mothers, the findings of our study provide preliminary evidence of an association between the mothers’ poor mental well-being and the psychological well-being of children, such as having behavioural difficulties. Evidence suggests that, because of maternal workload, children’s activities are often neglected, leading to children unintentionally engaging in negative activities (Schneider and Harknett, 2022; Das et al., 2023). Without parental guidance, children find it difficult to distinguish between good and bad behaviours, making them more vulnerable to accidents, behavioural problems and poor well-being (Han et al., 2010; Li and Chzhen, 2024). That is why it is important to ensure the well-being of working mothers to safeguard the well-being of their children.

### Strengthening institutional and community support systems

During the qualitative interviews, many participants suggested that an 8- to 9-h daycare facility, which is their working time, will significantly help the mental health of working mothers. A specific facility, where children will be taken care of, including ensuring nutrition, sleep and educational activities, will not only lessen the concerns of the mothers but will also ensure the children’s well-being (Behbehani et al., 2019; Behbehani et al., 2024). Both the tea garden management and the government can collaborate to provide childcare support, which includes daycare facilities, nutrition and education, for working mothers in the tea garden community.

The qualitative findings also identified the garden management, village council, government and NGOs as protective factors for the well-being of these women. Participants noted that in the tea garden community, girls marrying at a young age was common; however, thanks to awareness programmes by NGOs, this practice has declined compared to the past. Additionally, people of the tea garden community are now more inclined to educate their children rather than encourage them to work in the tea garden, breaking a long-standing generational cycle (Kamruzzaman et al., 2015). Support from garden management is crucial in ensuring a good working environment for these mothers, as the tea garden managers serve as a vital link between the workers and the management of the tea garden company (Shahadat and Uddin, 2022; Ahmed et al., 2022; Sinha, 2022). With cooperative management, women’s complaints about their work are listened to and addressed. Similarly, the village council also plays a vital role both at the workplace and at home. Participants repeatedly highlighted the strong advocacy of the village council, which strives to communicate with both the tea company management and the government. Therefore, strengthening the village council by providing operational and administrative support is essential to ensure that the concerns of working mothers are heard and actions are taken.

### Limitations

This study has several important limitations. First, the cross-sectional design limits the ability to infer causal relationships between maternal depression and child behavioural outcomes. Second, the use of purposive selection of tea estates and supervisor-provided sampling frames may have introduced selection bias, potentially affecting the representativeness of the sample. The purposive selection of tea gardens required management cooperation, which introduces gatekeeper influence at the site selection level, gardens with more progressive or cooperative management may differ systematically from those not included, limiting generalisability to the broader tea garden population in Bangladesh. Similarly, the sampling frames were prepared with input from garden supervisors, who may have introduced systematic bias in the lists of eligible mothers, despite cross-checking against village council records. These limitations should be considered when interpreting the findings.

Third, both maternal depression and child behavioural difficulties (SDQ parent version) relied on self-reported data, raising concerns about reporting bias. In particular, common method bias is a significant concern, as mothers experiencing psychological distress may be more likely to perceive and report their children’s behaviours negatively. This is especially relevant given the high proportion (87.8%) of abnormal scores observed in the parent-reported SDQ.

Additionally, the generalisability of the findings is limited to the four tea gardens included in the study and may not extend to other tea garden populations or similar settings. The cultural validity and contextual appropriateness of the measurement scales used, including the SDQ, remain uncertain in this specific population, which may have influenced the accuracy of the findings.

Finally, the qualitative component has limitations in representativeness. All FGD participants were permanent leaf pickers, and thus the perspectives of temporary workers, factory workers and other occupational groups represented in the quantitative sample were not captured. This imbalance limits the breadth of insights from the qualitative data.

## Conclusion

This study demonstrates a high burden of psychological distress among mothers working in tea garden communities in Bangladesh and identifies significant association with children’s behavioural and emotional well-being. Maternal mental health was significantly associated with multiple socio-demographic and occupational factors, including age, education, income, employment type and childcare arrangements. Qualitative findings further revealed that excessive workload, unsafe working conditions, financial insecurity, lack of childcare support and limited autonomy are perceived contributors maternal distress. While these findings highlight important relationships, the observational nature of this study limits causal interpretation. Future longitudinal and interventional research is needed to better understand the directionality and mechanisms underlying these associations. Additionally, addressing these challenges requires integrated interventions that combine workplace reforms, accessible childcare services, mental health awareness and strengthened social protection. Prioritising maternal mental health is essential to improving child well-being and reducing intergenerational vulnerability in marginalised tea garden communities.

## Supporting information

10.1017/gmh.2026.10251.sm001 Ansary et al. supplementary material Ansary et al. supplementary material

## Data Availability

All relevant analysed data are included within the manuscript and its Supplementary Files. Signed consent forms, audio recordings and fully coded transcripts are not publicly available due to ethical and confidentiality considerations. Access to these data may be granted upon reasonable request to the corresponding author, subject to approval by the participants.

## Supplementary material

The supplementary material for this article can be found at http://doi.org/10.1017/gmh.2026.10251.

## Long descriptions

### Table 1. Long description

The table consists of six columns and eleven rows of participant data.

1. I F 0 0 2: Male, Patrokhola Tea Garden, Shardar (Chief), 48 years old, 18 years experience.

2. I F 0 0 3: Male, Patrokhola Durga Temple, Temple caretaker, 45 years old, 20 years experience.

3. K M 0 0 4: Male, Sreemangal, Moulvibazar, Panchayat (village council) President, 58 years old, 25 years experience.

4. K M 0 0 5: Male, organisation not specified, Tea Garden manager, 46 years old, 20 years experience.

5. K M 0 0 6: Male, Horinganj Tea Garden, Head Teacher, 39 years old, 14 years experience.

6. K M 0 0 7: Male, Non-profit organisation in Sreemangal, Moulvibazar, Job holder, 60 years old, 32 years experience.

7. K M 0 0 8: Male, Sreemangal, Moulvibazar, Priest, 47 years old, 17 years experience.

8. K M 0 0 9: Female, Early Childhood Development (E C D), Teacher, 42 years old, 6 years experience.

9. I A O 1 0: Male, Family Planning Office, Women’s Division, Employee, 57 years old, 34 years experience.

10. I A 0 1 1: Female, Office of the Deputy Director, Women’s Affairs, Employee, 50 years old, 24 years experience.

11. I A 0 1 2: Male, Shidurkhan Tea Garden, Medical Dresser, 49 years old, 3 years experience.

Navigate back to Table 1..

### Table 2. Long description

The table contains 9 columns: Participants I D, Age in years, Gender, Marital Status, Number of Children, Occupation, Employment Status, Husband’s Occupation, and Experience in years. All participants are female and their occupation is picking leaves with permanent employment status.

Focus Group Discussion A includes 11 participants:

- A 1: 40 years, Widow, 1 child, Husband Labour, 3 years experience.

- A 2: 25 years, Married, 1 child, Husband Labour, 4 years experience.

- A 3: 25 years, Married, 1 child, Husband Labour, 2 years experience.

- A 4: 20 years, Married, 1 child, Husband Labour, 2 years experience.

- A 5: 21 years, Married, 1 child, Husband Labour, 3 years experience.

- A 6: 28 years, Married, 2 children, Husband Labour, 6 years experience.

- A 7: 25 years, Married, 1 child, Husband Labour, 2 years experience.

- A 8: 36 years, Married, 1 child, Husband Unemployed, 12 years experience.

- A 9: 24 years, Married, 1 child, Husband Labour, 2 years experience.

- A 10: 35 years, Widow, 1 child, Husband Labour, 7 years experience.

- A 11: 23 years, Married, 2 children, Husband Car driver, 6 years experience.

Focus Group Discussion B includes 10 participants:

- B 1: 25 years, Married, 1 child, Husband Unemployed, 2 years experience.

- B 2: 24 years, Married, 1 child, Husband Labour, 2 years experience.

- B 3: 24 years, Married, 2 children, Husband Labour, 2 years experience.

- B 4: 24 years, Married, 1 child, Husband Unemployed, 1 year experience.

- B 5: 25 years, Married, 1 child, Husband Labour, 3 years experience.

- B 6: 24 years, Married, 1 child, Husband Labour, 4 years experience.

- B 7: 38 years, Married, 1 child, Husband Unemployed, 10 years experience.

- B 8: 25 years, Married, 1 child, Husband Labour, 4 years experience.

- B 9: 27 years, Married, 3 children, Husband Unemployed, 15 years experience.

- B 10: 35 years, Married, 3 children, Husband Labour, 3 years experience.

Navigate back to Table 2..

### Table 3. Long description

The table presents demographic data across five columns: Variables, Categories, Number (N), Percentage (%), and Mean plus or minus S D.

- Age (years): Mean 29.24 plus or minus 5.04. Categories include 18 to 24 (11.8%), 25 to 34 (73.2%), 35 to 44 (13.7%), and 45 to 54 (1.3%).

- Educational qualification: None (25.3%), Primary (39.7%), Secondary (21.5%), and S S C / H S C or more (13.5%).

- Marital status: Married (96.9%), Separated (1.1%), and Widow (2.0%).

- Family type: Nuclear (62.7%) and Joint (37.3%).

- Number of children: Mean 1.9 plus or minus 0.89. Categories include One child (37.5%), Two children (41.9%), Three children (15.1%), and More than three children (5.6%).

- Occupation: Picking leaves (73.6%), Factory workers (2.4%), Temporary workers (12%), Garden workers (8.4%), and Others (3.5%).

- Work status: Permanent (35%) and Non-permanent (65%).

- Work hours: Mean 8.03 plus or minus 0.58.

- Work days: Mean 6.09 plus or minus 0.548.

- Income: Mean 8,701.4 plus or minus 3,716.3. Categories include Low-income less than 12,500 B D T (84%), Middle-income 12,500 to 21,000 (15.1%), and Upper income greater than 21,000 (0.7%).

Navigate back to Table 3..

### Table 4. Long description

The table consists of five columns: Descriptive statistics, Categories, N, percentage, and Mean plus or minus S D.

1. Depression level: Mean plus or minus S D is 11 plus or minus 6.2.

- Normal: 170, 37.7 percent.

- Mild: 109, 24.2 percent.

- Moderate: 146, 32.4 percent.

- Severe: 21, 4.7 percent.

- Extremely severe: 5, 1.1 percent.

2. Anxiety level: Mean plus or minus S D is 9.3 plus or minus 6.3.

- Normal: 179, 39.7 percent.

- Mild: 44, 9.8 percent.

- Moderate: 135, 29.9 percent.

- Severe: 60, 13.3 percent.

- Extremely severe: 33, 7.3 percent.

3. Stress level: Mean plus or minus S D is 18 plus or minus 7.5.

- Normal: 152, 33.7 percent.

- Mild: 81, 18 percent.

- Moderate: 144, 31.9 percent.

- Severe: 62, 13.7 percent.

- Extremely severe: 12, 2.7 percent.

4. Well-being: Mean plus or minus S D is 15.39 plus or minus 7.5.

- Poor: 153, 33.9 percent.

- Moderate: 185, 41 percent.

- Good: 113, 25.1 percent.

Navigate back to Table 4..

### Table 5. Long description

The table correlates demographic variables with levels of Depression, Anxiety, and Stress (Moderate, Severe, Extremely Severe) and Poor Well-being.

Key variables and significant P-values include:

* Age: Significant for Stress (0.014) and Well-being (0.002). The 25 to 34 age group has the highest frequency across all distress categories.

* Education qualification: Highly significant for Depression (less than 0.001) and Well-being (less than 0.001). Mothers with no or primary education show higher moderate to severe distress levels.

* Marital status: Significant for Depression (0.006) and Anxiety (0.022). Married mothers represent the vast majority of cases.

* Number of children: Significant for Anxiety (0.036). Mothers with two children show high moderate anxiety (42.2 percent).

* Occupation: Highly significant for Depression (less than 0.001), Anxiety (0.003), and Stress (less than 0.001). Leaf pickers are the most affected group, comprising approximately 75 to 81 percent of those with moderate to severe depression.

* Income: Significant for Anxiety (less than 0.001) and Well-being (0.001). Low-income mothers (less than 12,500 B D T) represent over 80 percent of all distress categories.

* Carer for children: Significant for Depression (less than 0.001), Stress (less than 0.001), and Well-being (0.026). Mothers relying on family members for childcare show the highest counts of moderate depression (69.9 percent).

Navigate back to Table 5..
