# Supplementary material for: Between leaves and lives: Maternal mental health among tea garden workers in Bangladesh and its association with children’s behavioural difficulties
Source: Glob Ment Health (Camb). 2026 Jun 15;13:e156. doi: 10.1017/gmh.2026.10251 (PMC13433331; doi:10.1017/gmh.2026.10251)
Supplement: Supplementary file 1 [file S2054425126102519sup001.pdf]

Supplementary file

Quantitative Questionnaire

Mental Health of Working Mothers in the Tea Garden Industry and Its Impact on Their Children's Well-Being

Section 1: Socio-demographics Information

1. Age	………… year
2. Educational Qualifications -	1. None<br>2. Primary<br>3. Secondary<br>4. SSC / HSC or Her More
3. Marital Status -	1. Single<br>2. Married<br>3. Separated<br>4. Divorced<br>5. Widow
4. Family Type -	1. Nuclear Family<br>2. Joint Family
5. Number of children and their ages	Number ………… Age ………
6. Main Work Type	1. Picking leaves<br>2. Factory Worker<br>3. Others
7. Work Day and hour in a week	Day ………… Hour ……………
8. Monthly Family Income	………………………………
9. When you are at work, who takes care of your child	1. Older Brother / Sister<br>2. Husband / Partner<br>3. Grandfather / Grandmother<br>4. Others Relative<br>5. Neighbors<br>6. Salaried Assistant<br>7. Take your children with you<br>8 ECDC / Day Care

Section 2: Depression, Anxiety and Stress Related Information

Please read each statement and circle a number 0, 1, 2, or 3, which indicates how much the statement applied to you over the past week. There are no right or wrong answers. Do not spend too much time on any statement.

**Questions**	**Did not apply to me at all (0)**	**Applied to me to some degree, or some of the time (1)**	**Applied to me to a considerable degree or a good part of the time (2)**	**Applied to me very much or most of the time (3)**
10. I found it hard to wind down				
11. I was aware of the drynes mouth				
12. I couldn't seem to experi positive feeling at all				
13. I experienced breathing difficulty (e.g. excessively rapid breathing, breathlessness in the absence of physical exertion)				
14. I found it difficult to work up the initiative to do things				
15. I tended to overreact to situations				
16. I experienced trembling (e.g. in the hands)				
17. I felt that I was using a lot of nervous energy				
18. I was worried about situations in which I might panic and make a fool of myself.				
19. I felt that I had nothing to look forward to				
20. I found myself getting agitated				
21. I found it difficult to relax				
22. I felt downhearted and blue				
23. I was intolerant of anything that kept me from getting on with what I was doing.				
24. I felt I was close to panic				
25. I was unable to become enthusiastic about anything				

26. I felt I wasn't worth much as a person				
27. I felt that I was rather touchy				
28. I was aware of the action of my heart in the absence of physical exertion (e.g. sense of heart rate increase, heart missing a beat)				
29. I felt scared without any good reason				
30. I felt that life was meaningless				

Section 3: Mental Well-being Related Information

Please respond to each item by marking one box per row, regarding how you felt in the last two weeks

**In the last two weeks**	**All the time**	**Most of the time**	**More than half the time**	**Less than half the time**	**Some of the time**	**At no time**
31. I have felt cheerful in good spirits.						
32. I have felt calm and relaxed.						
33. I have felt active and vigorous.						
34. I woke up feeling fresh and rested.						
35. My daily life has been filled with things that interest me.						

Section 4: Child's Physical Well-being Related Information

36. Has your child had frequent illness (fever, diarrhea, breathing problems) in the past 3 months?	1. Yes<br>2. Sometimes<br>3. No
37. Does your child get enough nutritious food daily (fruits, vegetables, milk, protein)?	1. Always<br>2. Sometimes<br>3. Never
38. How would you describe your child's sleep?	1. Good<br>2. Fair<br>3. Poor

Section 5: Child's Psychological Well-being Related Information (2-4 years old)

Questions	Not True	Somewhat True	Certainly True
1. Considerate of other people's feelings			
2. Restless, overactive, cannot stay still for long			
3. Often complains of headaches, stomach-aches or sickness			
4. Shares readily with other children (treats, toys, pencils etc.)			
5. Often has temper tantrums or hot tempers			
6. Rather solitary, tends to play alone			
7. Generally obedient, usually does what adults request			
8. Many worries, often seem worried			
9. Helpful if someone is hurt, upset or feeling ill			
10. Constantly fidgeting or squirming			
11. Has at least one good friend			
12. Often fights with other children or bullies them			
13. Often unhappy, downhearted or tearful			
14. Generally liked by other children			
15. Easily distracted, concentration wanders			
16. Nervous or clingy in new situations, easily loses confidence			
17. Kind to younger children			
18. Often argumentative with adults			
19. Picked on or bullied by other children			
20. Often volunteers to help others (parents, teachers, other children)			
21. Can stop and think things over before acting			
22. Can be spiteful to others			
23. Gets on better with adults than with other children			
24. Many fears, easily scared			

25. Sees tasks through to the end, good attention span			

Section 5: Child's Psychological Well-being Related Information (5-7 years old)

**Questions**	**Not True**	**Some what True**	**Certainly True**
1. Considerate of other people's feelings			
2. Restless, overactive, cannot stay still for long			
3. Often complains of headaches, stomach-aches or sickness			
4. Shares readily with other children (treats, toys, pencils etc.)			
5. Often has temper tantrums or hot tempers			
6. Rather solitary, tends to play alone			
7. Generally obedient, usually does what adults request			
8. Many worries, often seem worried			
9. Helpful if someone is hurt, upset or feeling ill			
10. Constantly fidgeting or squirming			
11. Has at least one good friend			
12. Often fights with other children or bullies them			
13. Often unhappy, downhearted or tearful			
14. Generally liked by other children			
15. Easily distracted, concentration wanders			
16. Nervous or clingy in new situations, easily loses confidence			
17. Kind to younger children			
18. Often lies or cheats			
19. Picked on or bullied by other children			
20. Often volunteers to help others (parents, teachers, other children)			
21. Thinks things out before acting			

22. Steals from home, school or elsewhere			
23. Gets on better with adults than with other children			
24. Many fears, easily scared			
25. Sees tasks through to the end, good attention span			

Qualitative Questionnaire

Research Questionnaire on Mental Health of Working Mothers in Tea Gardens - FGD

Overall mental health and emotional well-being

1. How is your emotional states (e.g. happy, anxious, tired, calm) in your daily life? Can you give an example of a recent experience?
2. What things make you feel good/empower you mentally?
3. Which things make you feel bad/weak, or uneasy?
4. What moments in your daily life bring you peace, happiness, or satisfaction?
5. Recently, have you had a time when you were very sad, anxious, or unable to function? Tell me about an incident. (Has it affected your work or family life?)
6. In what situation do you feel the most alone or helpless? What do you do then?

The impact of the workplace on mental health

1. What is a typical day at work like? Which parts of it are the most stressful (hard), and which parts are the most relaxing/satisfying?
2. What is your biggest challenge in managing work and family responsibilities?
3. How do you manage to take care of your children while you work?
4. Which aspect of family and work creates more stress? Why?
5. How much personal time or rest do you get because of work?
6. Which one change in managing work and family do you think would have reduced your stress the most?
7. What aspects of work give you satisfaction, pride, or strength?
8. What aspects of work create stress or emotional distress?
9. As a mother of a young child, how are you treated at work? (Probe: Respectful/ Bad/ Helpful)
10. Have you experienced any discrimination, harassment, or safety issues at work? (Share the incident) (If so, how did you deal with it?)
11. How is your body and mind after work?
12. Have you ever felt depressed or mentally exhausted because of work?

Family and social influences on mental health

1. How does your family view your work and stress?
2. How much do your spouse/children/relatives support you?
3. How do people usually react when you talk about mental health?

4. Have you ever heard or seen negative comments about working mothers in your community? If so, what kind?
5. If you seek help, how much support do you receive from family or society?
6. How much does your family help you emotionally or practically in managing work and childcare? Where do you find support, and where do you feel lonely?
7. What is the attitude towards working mothers in society? Does this attitude help your mental well-being, or create stress?

The impact of maternal mental health on child care, health, and development

1. How do you think your own emotions and stress affect your child's mental health or behaviour?
2. Have you noticed any changes in your child when you are stressed or sick?
3. Are there times when your child gives you the strength or happiness that helps you cope with stress?
4. How does your work impact childcare?
5. How much does work affect a child's study or play time?
6. Who takes care of the child in your absence?
7. What changes have you noticed in your child (such as decreased interest in play, decreased attention in studies, increased anger/fear) when you are under mental stress?
8. How does spending time with your child make you feel?
9. What kind of changes do you think would have happened in your child's life if the workload had been reduced?

Problem Dealing with Strategy

1. What types of ways do you use to cope with stress (e.g., family, friends, religious practice)?
2. What are your main obstacles to staying mentally healthy?
3. What things in life give you the most energy?
4. Have you ever felt like you need counseling or psychological help for yourself?
5. What kind of help would you like to make your life easier?

Effective action at the community and policy level

1. What community-level changes (such as childcare centers, mothers' groups, health care delivery) could make the biggest difference to your mental health and your child's well-being?
2. What kind of changes in workplace policies or government/NGO programs would be most beneficial for mothers like you?
3. What initiatives do you think are most important for mothers and young children in the tea garden community?

Research questionnaire on the mental health of working mothers in tea gardens (KII)

We are conducting a study on the mental health of working mothers in tea gardens and the impact on their children's well-being. Your experiences and opinions are very important to us in this study. You will be asked some questions, which may take approximately 30 minutes to answer. The information you provide will be used only for research purposes. It will not have any impact on your personal or professional life. Your name and personal identity will be kept completely confidential.

Perceptions of working mothers about mental health

- In your opinion, what is the mental health of mothers working in tea gardens like?
- In your opinion, what kind of mental problems do working mothers suffer from the most? What do you think are the main causes of these problems?
- What kind of impact do these problems have on their daily lives (family, workplace)?
- How much importance do you think families and society give to mental health?
- What are some common misconceptions or superstitions about mental health? If so, what are they?
- Do you think the rate of mental health problems has increased or decreased compared to the past? Why?
- What signs or behaviors have you noticed that indicate mothers are experiencing mental or emotional distress?
- Is there an open discussion about women's mental health in the community? Why is it or isn't it?

The impact of tea plantation work environment and administrative system

- Do you think the workplace environment affects women's mental health?
- How do work stress and safety affect mothers' mental health?
- In your experience, what are the main challenges that working mothers face in tea plantations (e.g.: overwork, wages, job insecurity, harassment, discrimination)?
- What kind of treatment do female employees receive from colleagues, supervisors, and management at work?
- What kind of support does the tea garden administration or management provide to reduce the mental stress of female workers?
- Are there any policies or benefits to protect women's mental health in the workplace?
- If so, how effective are they? Why? If not, what do you think is the reason?

How a mother's mental state affects a child's growth

- Do you think a mother's stress or depression is reflected in child care?
- How do you think this affects the child's physical health?
- Do you think a mother's work schedule affects her child's education?
- What positive changes do you think can happen in a child's life if the mother's mental health is good?
- Have you seen any specific examples where a child has been affected by a mother's stress?

Challenges and solutions to accessing mental health care

- Are there any mental health services or counseling facilities for tea garden workers? If so, how accessible and effective is it? If not, what are the gaps?
- What are the main barriers mothers face when seeking mental health support?
- How do families and society respond to their mental health problems?
- What kind of support system can be developed at the community level?
- What kind of initiatives do you think mothers would benefit the most from in the long run?
- What barriers are there in the current system (workplace, healthcare, education or social services) that prevent mothers from seeking mental health support?
- Are there any societal or cultural attitudes that discourage mothers from talking about or seeking help with mental health issues?
- In your opinion, what important services or support are missing here?

Community perspectives on mental health

- What kind of support do working mothers receive from family or neighbours in balancing work and child-rearing?
- What community norms or rules make it difficult for women to seek help for mental health?
- When a mother expresses stress or grief, what kind of reactions do people in the community typically show (e.g. support, neglect, ridicule, prejudice)? Do you know of any specific examples?"

Necessary steps for the well-being of mothers and children

- In your opinion, what should be the most urgent action to improve the mental health of working mothers—at work, in the family, and in the community?
- In your opinion, what role do tea garden authorities, local schools, and healthcare institutions play in providing psychological support to women?
- If you had the opportunity to make one change, what would it be and why?
- What changes at the workplace level do you think will help improve mothers' mental health and children's well-being?
- What changes at the policy level do you think could improve the mental health of tea garden workers and their children?
- What kind of role can the government, NGOs or tea garden authorities play in helping these mothers?
- If you could create a policy or program for working mothers in tea gardens, what would it be like?
