# Supplementary material for: Between leaves and lives: Maternal mental health among tea garden workers in Bangladesh and its association with children’s behavioural difficulties
Source: Glob Ment Health (Camb). 2026 Jun 15;13:e156. doi: 10.1017/gmh.2026.10251 (PMC13433331; doi:10.1017/gmh.2026.10251)
Supplement: Peer review history

# Reviewer report: Review: Between leaves and lives: Maternal mental health among tea garden workers in Bangladesh and its association with children’s behavioural difficulties — R0/PR2

**DOI:** 10.1017/gmh.2026.10251.pr2

**Authors:** Graham Powell

Intergenerational effects are neglected world wide so it was especially pleasing to see this research paper from a low/middle income country where it can be extremely difficult to break longstanding generational cycles of disadvantage.

The introduction touches on all the key issues related to the nature of the work, including poverty level remuneration, extreme physical demands, exploitation of marginalised groups, gender discrimination. This motivates the study and its aim to look at how this deprivation may impact upon the child.

The design and methods are simple and solid, using directly relevant and culturally valid psychometric tools and astute and thorough interview structures. The authors are to be congratulated on achieving a large sample size of 451 workers,

The results are meaningful and suggests where work needs to be done to ease the plight of the workers and the children. For example, it was very interesting to see how marriage was a burden, with husbands adding to the pressures and not sharing the workload or stresses. Society has a role in helping to change and shape marital expectations for the benefit of wives and ultimately for the benefit of the children. Such changes in role expectation are crucial if intergenerational cycles are to be broken.

There are indications in the paper of how progress can be made right now, the paper identifying a range of protective factors that potentially can be strengthened, including management of the gardens, the presence of effective village councils, the direct input from government and the input of NGOs.

This paper reinforces the need for targetted intervention and signposts what form such intervention might take.

# Reviewer report: Review: Between leaves and lives: Maternal mental health among tea garden workers in Bangladesh and its association with children’s behavioural difficulties — R0/PR3

**DOI:** 10.1017/gmh.2026.10251.pr3

Dear Authors, please find my detailed feedback aimed at refining and strengthening your manuscript for its next revision.

Title, abstract, and impact statement

• The title over-promises on “implications for child well-being” when the paper appears to include a limited child outcome assessment based mainly on SDQ classification and maternal depression comparison.

• The abstract is too general and lacks essential methodological detail. It should specify whether the study is cross-sectional, clarify how child well-being was operationalized, indicate the age range of children, and report key statistics more precisely. Phrases such as “high levels of psychological vulnerability” and “over 40% were vulnerable to poor psychological well-being” are vague unless the manuscript clearly defines what “vulnerable” means in relation to DASS-21 and WHO-5 categories. The abstract should report exact prevalence categories and the analytic approach linking maternal and child outcomes.

• The abstract states that qualitative data were obtained from “participants from Moulvibazar, Sylhet,” while the rest of the manuscript refers to tea gardens in Moulvibazar District and often specifically Sreemangal. The geographical description needs to be standardized throughout the paper. It is currently unclear whether “Moulvibazar, Sylhet” refers to district/division notation or whether the study setting spans multiple administrative labels inconsistently.

• The impact statement is too assertive relative to the reported evidence. Statements such as the study “demonstrates” significant association with adverse outcomes in children and “contributes to decolonizing global mental health research” require either stronger conceptual framing or softer language. The decolonizing claim, in particular, is normatively important but not analytically substantiated in the methods, reflexivity, authorship discussion, or participatory design description. The authors should either justify this claim explicitly or remove/soften it.

Introduction

• The introduction identifies a meaningful research gap, but the literature review needs sharper organization. At present, it moves between tea garden labor conditions, maternal mental health, general gendered labor burdens, and child outcomes without a clearly articulated conceptual framework linking these domains. The paper would benefit from an explicit framework showing hypothesized pathways from occupational precarity, household burden, and childcare arrangements to maternal distress, and from maternal distress to child behavioral difficulties.

• Some citations in the introduction appear weakly matched to the claims they support. For example, the sentence on women facing economic hardship and caregiving demands cites Hermes et al. (2010), which appears unrelated to common mental health burden in laboring mothers and instead concerns cardiovascular risk after hypertensive disorders in pregnancy. This is a notable citation mismatch and raises concern about reference quality control.

• The paper claims that “most existing studies on maternal mental health focus on women employed in professional or formal sector roles that primarily involve cognitive labour,” but this is too broad and likely inaccurate unless systematically supported. There is extensive maternal mental health literature in low-income, informal, and precarious labor contexts. The authors should narrow this claim, cite it more carefully, or rephrase it to note that this specific subgroup of tea garden workers remains under-studied rather than suggesting the broader field is mainly office-based.

• The introduction mixes “tea garden communities,” “tea garden industries,” “tea estates,” “tea garden settings,” and “a tea garden in the Moulvibazar region,” creating imprecision in the study scope. Since the sample was drawn from four tea gardens, the final objective statement should reflect that clearly. It should not say “a tea garden” if the study includes multiple estates.

Methods

• The description of the mixed-methods design is insufficiently specified. The authors should explicitly state whether this was a convergent, explanatory sequential, or exploratory sequential mixed-methods design. At present, the manuscript only says qualitative data were included “to capture nuanced perspectives and contextual factors,” which is not enough. The order of data collection, the integration strategy, and the rationale for mixing methods should be clearly stated.

• The manuscript says it used “a combination of purposive and stratified random sampling” guided by “operational feasibility,” and that tea estates were “purposely selected,” with eligible participants then identified from sampling frames provided by supervisors, village councils, and IDEA. This creates serious concerns about selection bias, gatekeeper influence, and representativeness. The authors need to explain exactly how the four tea gardens were chosen, how many eligible mothers existed in each garden, what the strata were, how randomization was implemented within strata, who prepared the list of eligible mothers, and whether any women refused participation or were excluded.

• The manuscript reports 451 mothers, but does not explain whether this number was based on prevalence estimation, power for group comparison, or logistical convenience. For the qualitative sample, 11 KIIs and 2 FGDs may be reasonable, but the authors should explain how they determined adequacy or saturation.

• For the quantitative component, the paper only states that mothers had at least one child aged 2–7 years. The authors should specify whether mothers had to be biologically related, primary caregivers, current workers, permanent or non-permanent workers, currently co-residing with the child, and whether mothers with more than one eligible child reported on the youngest, oldest, or a randomly selected child.

• The quantitative data were collected between 15 and 30 October 2025, whereas the FGDs were conducted on 23 and 24 September 2025. That ordering contradicts the abstract wording that qualitative data were obtained to “contextualize and deepen the statistical findings,” which implies that quantitative results came first. The paper should clarify the actual sequence and integration logic.

• The choice of DASS-21, SDQ, and WHO-5 is understandable, but the manuscript needs much stronger justification of measurement validity in this specific population. Tea garden workers are described as having low formal education and belonging largely to marginalized ethnic groups. The authors should state the language(s) used in administration, whether instruments were interviewer-administered, whether any cultural adaptation or cognitive testing was done, and how comprehension was ensured among low-literacy respondents.

• The manuscript does not report reliability for the scales in the present sample. Internal consistency values such as Cronbach’s alpha or omega should be provided for DASS-21 subscales, WHO-5, and SDQ total difficulties within this study sample.

• The DASS-21 scoring description is incomplete and slightly problematic. The authors report normal, mild, moderate, and extremely severe cut-offs, but omit severe cut-offs in the written description. That must be corrected because the manuscript later refers repeatedly to severe and extremely severe categories. Also, the phrase “shorter form of the DAS scale” is imprecise; it should clearly say DASS-21 scores were doubled to be comparable with DASS-42 conventions, if that is indeed what was done.

• The WHO-5 categorization needs justification. The paper uses three categories: poor, moderate, and good well-being, but WHO-5 is often discussed either as a raw score or transformed score, with common screening thresholds rather than a three-category classification. The authors should justify their chosen thresholds and indicate whether these thresholds are standard, locally validated, or adapted. Otherwise the language of “vulnerable to poor psychological well-being” remains somewhat arbitrary.

• The SDQ scoring description is insufficient. The manuscript says only items 6, 7, 14, and 21 were reverse-coded, but SDQ scoring depends on version, informant, age group, and subscale configuration. The authors should specify which version was used, who completed it, whether total difficulties excluded prosocial items, and whether the Bangladeshi norms/cut-offs used are validated for children aged 2–7, since SDQ application differs by age. This is especially important because the reported abnormal proportion of 87.8% is extraordinarily high and demands careful scrutiny of instrument choice and scoring.

• For qualitative tools, the manuscript is too general. It should summarize major domains included in KII and FGD guides, identify who conducted interviews, whether interviewers had prior relationships with participants, where interviews were held, their duration, and how privacy was maintained in communities where management involvement may create response inhibition.

• The phrase “descriptive statistics … while inferential tests … were conducted” is grammatically incomplete and methodologically vague. The authors should clearly specify which variables were continuous vs categorical, which tests were used for which comparisons, whether assumptions were checked, and what significance threshold was used. If multiple testing was extensive, some consideration of type I error should also be mentioned.

• Regression is mentioned but not explained. The paper must state what outcome(s) were modeled, what predictor variables were entered, whether models were linear, logistic, or ordinal, how multicollinearity was checked, how missing data were handled, and how variables were selected. If regression results were not ultimately used, the term should be removed from the Methods. This inconsistency between Methods and Results is a major reporting flaw.

• The paper does not describe missing data at all. The authors should report whether any items were missing in DASS-21, WHO-5, SDQ, or socio-demographic variables; how many cases were complete; and whether any imputation or listwise deletion was used.

• The qualitative analysis description also needs expansion. “Iterative coding, category development, and theme refinement” is too generic. The authors should describe whether coding was inductive, deductive, or hybrid; whether a codebook was developed; how disagreements were resolved; how much of the data were dual-coded; and what steps were taken to ensure reflexivity and credibility. “Two independent researchers coded a subset” is not enough without specifying the proportion or process.

• The authors mention triangulation, but there is no clear explanation of how qualitative themes were used to explain, corroborate, or expand quantitative patterns. They should state where integration occurred: design, methods, interpretation, or all three.

Results

• The manuscript needs a much clearer table-driven narrative. Many percentages are reported without denominators or confidence intervals.

• The terms “high levels,” “vulnerable,” “poor well-being,” and “vulnerable to poor psychological well-being” are not consistently defined in the text. For DASS-21, “high levels” appears to refer to severe/extremely severe combined, while “vulnerable” may refer to mild/mododerate or pre-severe states, but this is not explicitly stated. The same issue affects WHO-5. These category labels should be precisely defined in the Methods and consistently used in Results, Abstract, and Discussion.

• Several interpretations of group differences are potentially misleading because they rely on raw proportions in overwhelmingly large categories. For example, saying married participants constituted the majority across severity categories is not informative when 96.9% of the sample was married. This should not be interpreted as married status being substantively associated with distress unless the statistical comparison and category proportions within marital groups are presented appropriately. The current phrasing risks overstating the role of marital status.

• Similarly, the statement that participants aged 25–34 had higher proportions of stress and poor well-being may simply reflect that this group comprised 73.2% of the sample. The authors should present row or column percentages clearly and avoid statements based on absolute concentration within the largest category. This issue recurs for leaf pickers and mothers with two children.

• The sentence “Mothers faced extremely severe anxiety when they left their child with family members” is too strong and slightly awkward. The data do not show that mothers “faced” anxiety because of that arrangement; rather, the category of mothers who left children with family members had a higher proportion classified as extremely severe anxiety. The authors should rewrite this and provide the actual test statistic, degrees of freedom, and effect size.

• Reporting that 87.8% of children fell in the abnormal SDQ range is an extraordinary finding that demands careful validation, contextualization, and sensitivity analysis. Such a high prevalence may reflect scoring problems, instrument misapplication, informant bias, non-normative cut-offs, or a true but alarming phenomenon. The authors cannot simply report this figure without deeper interrogation. They should show subscale distributions, verify scoring, clarify informant/respondent procedure, and discuss whether the SDQ norms are appropriate for this age/context.

• The categories for child illness, nutrition, and sleep are poorly phrased and unclear. “Sometimes frequently ill” is logically inconsistent. Nutrition “sometimes” is not defined. Sleep results also appear inconsistent because 76.5% good plus 23.1% fair plus 4% poor exceeds 100%, unless there is a typographical error or overlapping categories.

• The inferential analysis linking maternal depression and child difficulties is under-reported. The manuscript states only that an independent-samples t-test showed greater child difficulty means among children of mothers with depression, p = 0.011. The authors must report group means, standard deviations, sample sizes, exact test statistic, degrees of freedom, and effect size. They should also explain how “mothers with depression” were defined: any non-normal DASS depression category? moderate or above? clinically meaningful threshold?

• Because both maternal depression and child SDQ are likely reported by the mother, common method bias is a serious concern. A distressed mother may perceive and report her child’s behavior more negatively. This limitation is must be acknowledged both in Results interpretation and in the Discussion/limitations.

• The qualitative findings section reads more like a thematic summary than a fully analyzed qualitative result. The manuscript should show more analytic depth: what is the relationship between themes, which were dominant across participant types, and where did KIIs and FGDs converge or diverge?

• The participant description for the qualitative sample needs polishing and correction. All FGD participants were permanent leaf pickers, which means the qualitative sample does not appear to capture the experiences of temporary workers, factory workers, or other occupational categories represented in the quantitative sample. This should be acknowledged as a limitation of qualitative representativeness.

• The KII composition is narrow and somewhat imbalanced. Only one healthcare worker and one management staff member were included, and the categories themselves are sometimes awkwardly labeled, for example “one healthcare worker (compounders)” and “two local government (municipal representatives).” The paper should explain why these stakeholder groups were selected and whether the sample was adequate to represent institutional perspectives.

• The quotations are vivid, but in several places the authors present themes as though they are community-wide realities without showing how widespread or contested they were across participants. For instance, claims about increasing or decreasing mental health awareness, exploitative quotas, or NGO withdrawal should be contextualized as reported perceptions unless corroborated by other data sources.

• The thematic structure could be tightened. Themes 2, 3, and 4 overlap substantially: workload, workplace adversity, and socioeconomic deprivation are analytically intertwined. The authors may wish to either retain separate themes but clarify their boundaries, or reorganize them into a more coherent hierarchy. As written, there is some repetition.

Discussion

• The Discussion contains multiple instances of over-interpretation. The statement “All participants with a high level were in the marriage group” is not analytically meaningful because nearly all participants were married. This kind of statement should be avoided unless it is normalized appropriately within group size. Otherwise it exaggerates the apparent importance of marriage.

• The Discussion often attributes mechanisms that were not directly tested quantitatively. For example, interpretations around husbands’ support, joint versus nuclear family living, menstrual strain, and maternal benefits stopping after the third pregnancy are plausible and interesting, but they should be clearly marked as qualitative explanations or hypotheses, not established statistical determinants unless measured and analyzed quantitatively.

• The statement that lower-income mothers were more prone to anxiety and middle-income mothers had better well-being needs direct statistical support. The paper should present the actual coefficients or cross-tabulated proportions in the Results. Right now the Discussion is more confident than the Results section permits.

• The manuscript also mixes quantitative evidence and anecdotal quotations without clear boundaries. For example, exploitative wage withholding, quotas, toilets, thirst, menstruation, and lack of shelters are discussed as if they are core quantitative findings, yet they appear to be qualitative insights. The Discussion should distinguish clearly between findings derived from measured survey variables and those emerging from qualitative narratives.

• The subsection on correlation between mother’s and child’s psychological well-being should be more cautious. A single t-test on child difficulties by maternal depression status does not establish a broader maternal-child mental health mechanism. The current wording implies a robust intergenerational pathway, but the analysis shown is limited. It should be reframed as preliminary evidence of association.

• The daycare recommendation is reasonable, but the paper should avoid claiming that an 8- to 9-hour daycare facility “will significantly help” maternal mental health unless this was directly evaluated. The qualitative data support participant preference for childcare support; they do not demonstrate intervention effectiveness in this setting.

• The authors should add a dedicated limitations paragraph or section addressing: cross-sectional design, potential selection bias from purposive estate selection and supervisor-provided sampling frames, self-report bias, common method bias in maternal-child outcome linkage, limited generalizability beyond four tea gardens, unclear cultural validity of scales in this population, possible misclassification in SDQ scoring, and qualitative sample imbalance.

Conclusion

• The conclusion still overstates the evidence in places. Saying maternal mental health “was influenced by” age, education, income, employment type, and childcare arrangements is too causal. It should say “was associated with” these factors. Similarly, the link to child well-being should be described as significant association, not impact or effect.

• The conclusion would be stronger if it briefly acknowledged the observational nature of the study and called for longitudinal or intervention research rather than ending only with policy prescriptions.

# Editor report: Recommendation: Between leaves and lives: Maternal mental health among tea garden workers in Bangladesh and its association with children’s behavioural difficulties — R0/PR4

**DOI:** 10.1017/gmh.2026.10251.pr4

**Affiliations:** Clinical Psychology, National Institute of Mental Health and Neuro Sciences Department of Clinical Psychology, India

Dear Authors,

I am happy to share with you that your manuscript has received blinded reviews. I hope you will find the comments and suggestions useful to improvise the manuscript. I look forward to receiving the revised manuscript. 

Thank you for choosing Cambridge Prisms: Global Mental Health to showcase your work.

Sincerely,

Thomas

# Editor report: Decision: Between leaves and lives: Maternal mental health among tea garden workers in Bangladesh and its association with children’s behavioural difficulties — R0/PR5

**DOI:** 10.1017/gmh.2026.10251.pr5

**Affiliations:** Johns Hopkins University Bloomberg School of Public Health, United States

No accompanying comment.

# Reviewer report: Review: Between leaves and lives: Maternal mental health among tea garden workers in Bangladesh and its association with children’s behavioural difficulties — R1/PR7

**DOI:** 10.1017/gmh.2026.10251.pr7

I am satisfied with the revisions the authors made and recommend to accept the manuscript in its current form.

# Editor report: Recommendation: Between leaves and lives: Maternal mental health among tea garden workers in Bangladesh and its association with children’s behavioural difficulties — R1/PR8

**DOI:** 10.1017/gmh.2026.10251.pr8

**Affiliations:** Clinical Psychology, National Institute of Mental Health and Neuro Sciences Department of Clinical Psychology, India

Dear Authors,

I am happy to share with you that the independent reviewers are satisfied with the revised manuscript. You will hear further about the progress of the manuscript from the journal office. 

Thank you for submitting your work to the Cambridge Prisms: Global Mental Health. I hope you will consider this journal for your future work.

With best wishes,

Thomas

# Editor report: Decision: Between leaves and lives: Maternal mental health among tea garden workers in Bangladesh and its association with children’s behavioural difficulties — R1/PR9

**DOI:** 10.1017/gmh.2026.10251.pr9

**Affiliations:** Johns Hopkins University Bloomberg School of Public Health, United States

No accompanying comment.
